# Transversus Abdominis Plane Block Following Cesarean Section: A Prospective Randomized Controlled Study Comparing the Effects on Pain Levels of Bupivacaine, Bupivacaine + Dexmedetomidine, and Bupivacaine + Dexamethasone

**DOI:** 10.3390/jcm13144270

**Published:** 2024-07-22

**Authors:** Senem Urfalı, Sedat Hakimoğlu, Selim Turhanoğlu, Onur Koyuncu

**Affiliations:** Department of Anesthesiology and Reanimation, Tayfur Ata Sokmen Medical Faculty, Hatay Mustafa Kemal University, 31040 Hatay, Turkey; sedathakimoglu@gmail.com (S.H.); adat63@gmail.com (S.T.); dronurkoyuncu@gmail.com (O.K.)

**Keywords:** bupivacaine, dexmedetomidine, dexamethasone, postoperative analgesia, cesarean section

## Abstract

**Background**: The transversus abdominis plane (TAP) block is providing effective postoperative analgesia in patients undergoing cesarean section (CS). This study aims to evaluate and compare the effects on pain levels of bupivacaine alone versus bupivacaine combined with dexmedetomidine and bupivacaine combined with dexamethasone in ultrasound-guided TAP block for postoperative pain after CS. **Material and Method**: In this randomized controlled trial, 120 patients with American Society of Anesthesiologists (ASA) physical status I and II scheduled for elective cesarean section under spinal anesthesia were randomly divided into three groups. At the end of the surgery, an ultrasound-guided TAP block was performed on all patients: bupivacaine 0.5% (Group B), bupivacaine 0.5% + dexmedetomidine (1 µg/kg) (Group BD), and bupivacaine 0.5% + dexamethasone (4 mg) (Group BDx). Postoperatively, all patients were evaluated at 0, 1, 4, 8, 16, and 24 h for visual analog scores VASs, tramadol consumption, complications, and patient satisfaction. A p value of < 0.05 is statistically significant. **Results**: At 0 h, VASs in the sitting and supine positions were significantly higher in the BDx group (0.85 ± 1.61 and 0.85 ± 1.36, respectively) compared to the B group (0.05 ± 0.32 in both positions) and the BD group (0.15 ± 0.48 in both positions) (*p* = 0.005 and *p* = 0.001, respectively). At the 24th hour, VASs in the sitting and supine positions were significantly lower in the BDx group (1.7 ± 1.2 and 1.43 ± 1.05) compared to the B group (2.3 ± 0.68 and 2.2 ± 0.72) and the BD group (2.57 ± 1.01 and 2.28 ± 0.78) (*p* = 0.005 and *p* = 0.001, respectively). At 0 h, the tramadol requirement was highest in the BDx group at 12.5%, while it was not required in the B and BD groups (*p* = 0.005). At 0 h, the rate of nausea and vomiting was highest in the BDx group at 17.5%, compared to 2.5% in the BD group and 0% in the B group (*p* = 0.003). Patient satisfaction scores were higher in the dexamethasone group compared to the other groups. This was significant between Group B and Group BDx (*p* = 0.009 < 0.05). **Conclusions**: Adding dexmedetomidine or dexamethasone to bupivacaine in ultrasound-guided TAP blocks reduces postoperative pain and increases patient satisfaction after cesarean sections. Dexamethasone, due to its delayed onset but extended duration, achieves lower pain scores and higher satisfaction. Further research is necessary to confirm these findings.

## 1. Introduction

Cesarean section (CS) is one of the most common surgeries and usually causes moderate to severe pain for up to 48 h [1]. The rate of CS, which is a life-saving surgical procedure in cases of certain complications that occur during pregnancy and birth, has exceeded 20% worldwide [2,3]. Pain control after CS has crucial importance, especially in the first 24 h, to facilitate early ambulation and the establishment of breastfeeding [4]. Insufficient analgesia in the postoperative period may cause a number of undesirable effects, such as patient discomfort, thromboembolism due to extended immobilization, and decreased pulmonary clearance resulting in complications [5].

Postoperative CS pain arises mainly from somatic and visceral components. Surgical incision of the tissue leads to somatic pain, while visceral pain is mainly associated with inflammation [6]. The transversus abdominis plane (TAP) block has gained increasing importance as a very effective pain relief in abdominal surgery since Rafi’s application in 2001 [7]. The transversus abdominis plane (TAP) block comprises the administration of a local anesthetic agent between the transverse and internal oblique muscles of the abdominal wall, targeting spinal T7-L1 levels, which attenuates somatic pain by inhibiting neural afferents within the transversus abdominis plane [4,8]. This reduces postoperative pain from lower abdominal surgeries, including cesarean sections [9]. Although commonly used nonsteroidal anti-inflammatory drugs and paracetamol can only supplement other modes of analgesia, they are not sufficient for their own population. In this context, many studies are being conducted to increase the TAP blockage duration [10].

A variety of anesthetic agents such as bupivacaine, levobupivacaine, and ropivacaine are commonly used in TAP blocks [9]. These drugs provide short-term analgesia and primarily alleviate somatic pain [11,12]. To extend the duration of analgesic efficacy, various adjuvants such as opioids, ketamine, clonidine, and alpha-2 agonists like dexmedetomidine have been added [9]. Studies have consistently shown that optimizing analgesia reduces opioid consumption and alleviates postoperative symptoms such as nausea and sedation [1].

Bupivacaine is one of the widely used local analgesics [13]. Dexmedetomidine is a highly selective central alpha-2 adrenergic agonist. It strengthens local anesthetic effects and prolongs analgesic duration by blocking the transmission of nerve signals through C and A delta fibers [4,14]. For these reasons, dexmedetomidine is commonly used as an adjuvant to local anesthetics in many studies [4,5,15]. Dexamethasone, a corticosteroid, provides analgesia owing to its anti-inflammatory properties. It also potentiates the effect of local anesthetics [16]. In light of this knowledge, dexamethasone has been used as an adjuvant in various studies to improve the anesthetic effects of TAP block postoperative CS operations [1,4].

Recent studies have highlighted the potential of non-opioid analgesic techniques in managing various pain conditions. For example, laser therapy has proven effective in alleviating chronic headache symptoms by targeting mucosal contact points in the nasal cavity [17]. Similarly, fractional CO_2_ laser technology has provided significant relief for patients with Lichen Sclerosus, offering a non-invasive alternative that improves histological outcomes and reduces discomfort without requiring general anesthesia [18]. These advancements underscore the importance of exploring non-opioid treatments, such as TAP blocks and laser therapy, alongside adjuvants, particularly in mitigating postoperative pain following cesarean sections.

This study aimed to assess and compare the effects on pain levels of bupivacaine alone versus bupivacaine combined with dexmedetomidine and bupivacaine combined with dexamethasone in an ultrasound-guided TAP block for managing postoperative pain after cesarean section. Our primary outcome was pain score, while the secondary outcomes were analgesic requirements, complications, and patient satisfaction. 

## 2. Materials and Methods

### 2.1. Study Design

The study was conducted between February 2020 and September 2021 at Hatay Mustafa Kemal University, following the approval from the Clinical Research Ethics Committee (meeting date: 20 February 2020, decision no: 05). Written informed consent was obtained from all participants in accordance with the principles of the Declaration of Helsinki [19]. 

Pregnant women with an American Society of Anesthesiologists (ASA) score of I or II who were scheduled for cesarean section under spinal anesthesia were included in the study. The exclusion criteria were as follows:

- Refusal to participate in the study;
- Known allergies to local anesthetics/opioids/nonsteroidal anti-inflammatory drugs;
- Presence of infection at the needle entry site for the block;
- Recent use of glucocorticoids;
- Diabetes mellitus;
- Pregnancy-induced hypertension;
- Use of chronic pain medications;
- Body mass index (BMI) > 35.

In the operating room, electrocardiography, pulse oximetry, and noninvasive blood pressure were monitored for all patients from the beginning of the procedure until they left the operating room. After monitoring the patients, spinal anesthesia was administered in a seated position with 2 to 2.2 mL of 0.5% heavy bupivacaine (Marcaine^®^, Astra-Zeneca, Istanbul, Turkey) between the L3 and L4 vertebrae after confirming the proper flow of cerebrospinal fluid. 

Subjects included in the study were randomly assigned to three groups using a computer-generated sequence of random numbers, regardless of their demographic characteristics.

Patients were equally (40 per group) allocated to three TAP block groups as follows:

1. Group B: Bupivacaine 0.5% 10 mL + physiological saline 10 mL;
2. Group BD: Bupivacaine 0.5% 10 mL + physiological saline 9 mL + dexmedetomidine 1 mL (1 µg/kg);
3. Group BDx: Bupivacaine 0.5% 10mL + physiological saline 9 mL + dexamethasone 1mL (4 mg).

At the end of the surgery, in all patients, a single-injection, ultrasound-guided (SonoSite M-Turbo) Transversus Abdominis Plane (TAP) block was administered utilizing a linear ultrasound probe (12 MHz). With the patient positioned supine, the ultrasound probe was placed transversely on the lateral abdominal wall, in the midaxillary line, between the lower edge of the ribcage and the iliac crest. The needle was inserted in-plane directly beneath the ultrasound probe and advanced until it reached the layer between the internal oblique and transversus abdominis muscles. Once this layer was reached, the local anesthetic (LA) solution was injected, causing the TAP to expand and appear as a hypoechoic area. To prevent vascular puncture, careful aspiration was performed before the injection. The procedure was then repeated on the contralateral side using the same technique. Post-surgery, patients were transferred to the post-anesthesia care unit. 

Patients transferred to the post-anesthesia care unit were routinely given paracetamol (1 mg) intravenously for 24 h, every 6 h. Visual analog scores (VASs) were evaluated at 0, 1, 4, 8, 16 and 24 h. VASs were evaluated in both sitting and supine positions. Pain levels were assessed using visual analog scores (VASs), with scores of 2–4 considered mild pain, 5–7 considered moderate pain, and 8–10 considered severe pain [20]. Those with a VAS ≥ 4 were given 0.5 mg/kg tramadol intravenously.

Bradycardia was defined as HR < 50 bpm and was treated with an intravenous injection of 0.5 mg of atropine. Hypotension was defined by a mean arterial pressure decrease of more than 30% from baseline and was treated with a 200 mL fluid bolus and a 5 mg intravenous injection of ephedrine. Nausea and vomiting were assessed using a 4-point scale (0 = none, 1 = nausea, 2 = retching, 3 = vomiting). Patients who scored ≥1 were treated with 4 mg of IV ondansetron. 

Instant and total analgesic consumption and complications were intermittently recorded during the first 24 h postoperatively. Patient satisfaction was evaluated after 24 h. 

#### Statistical Method

In this study, which was designed as an active Randomized Controlled Trial, patients who underwent cesarean section were randomized into three treatment groups: Bupivacaine (Group B), Bupivacaine + Dexmedetomidine (Group BD), and Bupivacaine + Dexamethasone (Group BDx). The primary outcome variable, VAS scale scores, was compared between treatment groups. Based on a minimum clinically significant mean difference of 0.2 on the VAS scale (Cohen’s d effect size) and allowing for a maximum type I error of 5% and a minimum power of 80%, the sample size for each group has been determined to be 40 patients.

Statistical analysis of the data was performed using IBM SPSS Version 21 and the MedCalc statistical package program. Due to the suitability of the Central Limit Theorem, parametric tests were used for continuous measurements without normality testing [21]. However, since VAS and Satisfaction Scores were ordinal variables, non-parametric testing was used. When making statistical analysis of continuous data on scales, mean ± standard deviation, median, and 25%-75% values are used. Frequency and percentage values were used to describe categorical variables.

The One-Way ANOVA test and Kruskal–Wallis H statistics were used to compare continuous measurement averages in three groups. If a difference was detected, pairwise comparisons were evaluated with Tukey statistics as a post hoc test. The chi-square test was used to evaluate the relationship between categorical variables. If a relationship was detected, z-test statistics were used to evaluate the difference in pairwise ratio.

**For statistical significance, *p* < 0.05 was accepted.**

## 3. Results

A total of 120 pregnant women, aged between 18 and 42 years (with a mean age of 30.1 ± 5.6), scheduled to undergo cesarean section under spinal anesthesia were included in the study. The relationship between socio-demographic and operational characteristics is presented in Table 1. There was no significant difference between the groups in terms of mean age (*p* * = 0.23 > 0.05). The ASA score was 1 in 48.3% of the patients and 2 in 51.7%, with no significant differences between the groups in terms of ASA status (*p* * = 0.85 > 0.05). BMI values were 29.41 ± 2.89 for Group B, 29.03 ± 2.98 for Group BD, and 27.62 ± 2.34 for Group BDx, with significant differences observed between the groups (*p* * = 0.01 < 0.05). This significance was particularly notable between Group B and Group BDx (*p* ** = 0.01 < 0.05). While the operating time (minute) was 77.1 ± 17.7 in Group B and 68 ± 11.6 in Group BD, it was lower in Group BDx, at 66.8 ± 14.8. There was a significant difference between the mean operation times according to the groups (*p* * = 0.004 < 0.05). This difference was between Group B and Group BD, and Group B and Group BDx (*p* ** = 0.02, 0.007 < 0.05).

The mean block time (in minutes) was also a significant difference between the groups (*p* * = 0.001 <0.05). This difference was between Group B and Group BD, and Group B and Group BDx (*p* ** = 0.001, 0.002 < 0.05). The mean block times were 10.9 ± 2.4 in Group B, 9.1 ± 1.5 in Group BD, and 9.2 ± 2.4 in Group BDx.

As explained in Table 2, the 0-h instant analgesic tramadol requirement was 12.5% in the BDx group, it was not required in the B and BD groups (*p* * = 0.005 < 0.05). The difference was also significant at the 1st hour [between Group B and Group BDx (*p* ** = 0.01 < 0.05), and Group BD and Group BDx (*p* ** = 0.003 < 0.05)] and at the 8th hour [between Group B and Group BD (*p* ** = 0.008 < 0.05), and Group BD and Group BDx (*p* ** = 0.03 < 0.05)], however no significant differences were observed at the 4th, 16th, and 24th hours. The 0-h total analgesic tramadol requirement was 12.5% in the BDx group, it was not required in the B and BD groups. This difference between the groups was significant (*p* * = 0.005 < 0.05). While this difference was significant at the 1st hour between Group B and Group BDx (*p* ** = 0.001 < 0.05), and Group BD and Group BDx (*p* ** = 0.001 < 0.05) and 4th hour between Group B and Group BDx (*p* ** = 0.004 < 0.05), and Group BD and Group BDx (*p* ** = 0.01 < 0.05), there was no significant difference at the 8th, 16th and 24th hours.

At hour 0, there was a significant relationship between the groups regarding the incidence of nausea and vomiting (*p* * = 0.003 < 0.05). The rates of nausea and vomiting in group 3 were significantly different compared to groups 1 and 2 (*p* ** = 0.006, 0.03 < 0.05). Additionally, at hour 0, there was a significant relationship between the groups concerning the occurrence of complications (*p* * = 0.009 < 0.05). The complication rates in group 3 were significantly different compared to groups 1 and 2 (*p* ** = 0.01, 0.04 < 0.05). However, at hours 1, 4, 8, 16, and 24, there was no significant relationship between the three groups regarding nausea, vomiting, and complications (*p* * > 0.05). At hours 1, 4, 8, 16, and 24, there was no significant relationship between the three groups regarding antiemetic usage (*p* * > 0.05).

Headache, hypotension, bradycardia, arrhythmia, tinnitus, and numbness around the mouth were not observed in all three groups.

In Table 3, there was a significance at hour 1 in terms of instant analgesic amount (*p* * = 0.04 < 0.05). The average amounts of tramadol used were 45 ± 0, 40 (only one patient), and 37.2 ± 3.67 mg in Group B, Group BD and Group BDx, respectively. In the comparison between groups for hour 1, the difference between Group B and Group BDx was significant (*p* ** = 0.04 < 0.05). No significant relationship was observed at other follow-up hours for instant analgesic tramadol usage amount (*p* * = 0.6, 0.06, 0.22, and 0.27 > 0.05 for the 4th, 8th, 16th, and 24th hours, respectively).

Significant differences in total tramadol consumption were observed at the 8th hour (*p* * = 0.003 < 0.05); 55.47 ± 19.81 mg for Group B, 41.13 ± 11.16 mg for Group BD, and 65.33 ± 26.04 mg for Group BDx and at the 16th hour (*p* * = 0.04 < 0.05); 77.97 ± 31.31 mg for Group B, 55.57 ± 19.38 mg for Group BD, and 71.78 ± 34.96 mg for Group BDx. At the 8th hour, a significant difference was observed between Group BD and Group BDx (*p* ** = 0.002 < 0.05). At the 16th hour, the significant difference was between Group B and Group BD (*p* ** = 0.04 < 0.05). No significant differences in total tramadol consumption were found at other follow-up times (*p* * = 0.86, 0.39, and 0.18 for the 1st, 4th, and 24th hours, respectively).

Evaluation according to the visual analog score (VAS) was presented in Table 4. In the sitting position, the difference in mean VASs between the groups was significant at hour 0 (*p* * = 0.005 < 0.05). These scores were significant between Group B and Group BDx as well as in Group BD and Group BDx (*p* ** = 0.001, 0.005 <0.05, respectively). In Group B, the mean VAS was 0.05 ± 0.32, with a median and quartiles of 0 (0–0). In Group BD, the mean VAS was 0.15 ± 0.48, with a median and quartiles of 0 (0–0). In Group BDx, the mean VAS was 0.85 ± 1.61, with a median and quartiles of 0 (0-2). The mean VASs between the groups were also significant at the 24th hour (*p* * = 0.005 < 0.05). These scores were significant between Group B and Group BDx as well as between Group BD and Group BDx (*p* ** = 0.03, 0.001 < 0.05 respectively). In Group B, the mean VAS was 2.3 ± 0.68 with a median and quartiles of 2 (2–3). In Group BD, the mean VAS was 2.57 ± 1.01, with a median and quartiles of 2 (2–3); and in Group BDx, the mean VAS was 1.7 ± 1.2, with a median and quartiles of 1 (1–3). There were no significant differences at other follow-up hours.

At the supine position, the difference in mean VASs between the groups was significant at 0 h (*p* * = 0.001 < 0.05). These scores were significant between Group B and Group BDx as well as between Group BD and Group BDx (*p* ** = 0.001, 0.001 < 0.05, respectively). In Group B, the mean VAS was 0.05 ± 0.32, with a median and quartiles of 0 (0–0); in Group BD, the mean VAS was 0.15 ± 0.48, with a median and quartiles of 0 (0–0); and in Group BDx, the mean VAS was 0.85 ± 1.36, with a median and quartiles of 0 (0–0). Similarly, the VASs between the groups were also significant at the 24th hour (*p* * = 0.001 < 0.05) (*p* ** = 0.001 and 0.001 < 0.05 for Group B and in Group BD as well as in Group B and Group BDx, respectively). In Group B, the mean VAS was 2.2 ± 0.72 with a median and quartiles of 2 (2–3); in Group BD, the mean VAS was 2.28 ± 0.78 with a median and quartiles of 3 (2–3); and in Group BDx, the mean VAS was 1.43 ± 1.05 with a median and quartiles of 2 (1–3.5). There were no significant differences at other follow-up hours.

The difference between the mean satisfaction scale was significant (p* = 0.02 < 0.05) These scores were significant between Group B and Group BDx (p** = 0.009 < 0.05). In Group B, the mean satisfaction scale was 2.73 ± 0.98 with a median and quartiles of 3 (2–3); in Group BD, the mean satisfaction scale was 3.05 ± 0.78 with a median and quartiles of 3 (3–4); and in Group BDx, the mean the satisfaction scale was 3 ± 0.79 with a median and quartiles of 3 (3–4) (Table 4).

## 4. Discussion

Cesarean section is one of the major surgical procedures and, as expected, there is significant postoperative discomfort and pain [22]. Post-cesarean analgesia aims to balance maternal comfort and newborn safety, but opioids, despite their effectiveness, can cause side effects like nausea, vomiting, and dizziness, reducing patient satisfaction [11,22,23]. Hence, various drugs or techniques such as non-opioids, adjuvants, and TAP block have been used to reduce perioperative opioid consumption and thus its adverse effects [24]. The TAP block, beneficial for pain management during cesarean delivery, shares similarities with minimally invasive laparoscopic techniques that aim to reduce surgical pain and enhance recovery [25]. An ideal local anesthetic should provide complete sensory blockade and have an optimal duration of action. Among commonly used local anesthetics, bupivacaine has a significantly longer duration of action compared to lidocaine and ropivacaine [13,26]. Adding adjuvants to local anesthetics prolongs postoperative analgesia and decreases the necessity for rescue analgesics [27,28]. Recent studies indicate that adding adjuvants such as dexmedetomidine and dexamethasone into TAP block effectively lowers pain scores and reduces the need for additional analgesics while keeping side effects minimal [11,29,30]. A study found that the time to first rescue analgesia was significantly longer with ropivacaine plus dexamethasone compared to ropivacaine alone (19.04 ± 4.13 h vs. 11.62 ± 3.80 h; *p* < 0.001) [27]. Another study showed lower tramadol consumption in the bupivacaine plus dexamethasone group compared to bupivacaine alone (50.0 ± 35 mg vs. 92.9 ± 36 mg; *p* < 0.001) [28]. McDonnell et al. reported that pregnant women receiving a TAP block with ropivacaine had over a 70% reduction in morphine requirements and a delayed time to the first PCA morphine request compared to the placebo group. Additionally, postoperative VAS pain scores, both at rest and during movement, were lower in the TAP block group [22].

Ramya et al. reported in their study that the block duration was 14 h in the bupivacaine + dexmedetomidine TAP block and 8 h in the bupivacaine TAP block alone [15].

While Sachdeva et al. found that the combination of dexamethasone and ropivacaine significantly extended the time to the first analgesic requirement (5.92 vs. 3.11 h) and reduced postoperative tramadol use (100 mg vs. 140 mg) compared to ropivacaine alone, these findings do not align with our study. However, the improvement in patient satisfaction (57.14% vs. 25.71%) is consistent with our results [1].

In the study by Thakur et al., where dexmedetomidine and dexamethasone were added to bupivacaine in TAP block during cesarean sections, similar to our findings, higher pain levels and analgesic requirements were observed in the dexamethasone group during the initial hours [31]. In another study by Sinha et al., where dexamethasone and bupivacaine were added to levobupivacaine in the TAP block during hysterectomies, similar to our study, higher VASs and shorter time to the first rescue analgesia were observed in the dexamethasone group within the first hour. However, unlike our study, the patient satisfaction scale was higher in the dexmedetomidine group [32]. We hypothesize that the higher VASs and analgesic requirements observed in the dexamethasone group within the first hour are due to the delayed onset of action of dexamethasone. Additionally, Sinha et al. observed higher rates of nausea and vomiting in the dexmedetomidine group at the end of 24 h. In contrast, we found higher rates of nausea and vomiting in the dexamethasone group within the first hour [32].

The differences in results could also be due to variations in study designs, patient demographics, and dosages administered. For instance, a higher body mass index (BMI) in certain patient groups, as observed in our study, may influence the distribution and effectiveness of the local anesthetics and adjuvants.

## 5. Study’s Limitations

This study is limited by its single-center design, which may affect the results. A larger, multi-center study is recommended to validate these findings across different populations. Additionally, the study only evaluated analgesia and associated outcomes within the first 24 h postoperatively. Longer follow-up periods are necessary to assess the sustained efficacy and potential long-term impacts of the interventions.

## 6. Conclusions

The addition of dexmedetomidine or dexamethasone to bupivacaine for ultrasound-guided TAP blocks improves postoperative analgesia and patient satisfaction in cesarean section patients. Dexamethasone as an adjuvant appears to be beneficial, providing lower pain scores and higher satisfaction levels with a late onset but long-lasting effect. Further research with follow-up periods is suggested to confirm these findings and optimize postoperative pain management strategies.

## Figures and Tables

**Table 1:** Socio-demographic characteristics, operation, and block times of the groups (n = 120).

	Bupivacaine (n = 40)	Bupivacaine +Dexmedetomidin (n = 40)	Bupivacaine + Dexametazon (n = 40)		
	Mean ± SD	Mean ± SD	Mean ± SD	*p* Value *	*p* Value ** 1 vs. 2, 1 vs. 3, 2 vs. 3
Age	30.1 ± 5.4	31.5 ± 5.9	28.9 ± 5.2	0.23	−
BMI	29.41 ± 2.89	29.03 ± 2.98	27.62 ± 2.34	**0.01**	0.82, **0.01**, 0.06
ASA score	**n (%)**	**n (%)**	**n (%)**		
I	19 (47.5)	18 (45)	21 (52.5)	0.85	−
II	21 (52.5)	22 (55)	19 (17.5)		
Operation time	77.1 ± 17.7	68 ± 11.6	66.8 ± 14.8	**0.004**	**0.02**, **0.007**, 0.93
Block time	10.9 ± 2.4	9.1 ± 1.5	9.2 ± 2.4	**0.001**	**0.001**, **0.002**, 0.91

* One-Way ANOVA test|Chi-square test, ** Post Hoc test-Tukey; *p* < 0.05 is significant. BMI: body mass index above, ASA: American Society of Anesthesiologists, SD: Standard deviation.

**Table 2:** Assessment of clinical relationship between groups (n = 120).

	Bupivacaine (n = 40)	Bupivacaine +Dexmedetomidin (n = 40)	Bupivacaine + Dexametazon (n = 40)		
Hour	n (%)	n (%)	n (%)	*p* Value *	*p* Value ** 1 vs. 2, 1 vs. 3, 2 vs. 3
**Instant analgesic** **tramadol requirement**					
0	0 (0)	0 (0)	5 (12.5)	**0.005**	−**0.02**, **0.02**
1	2 (5)	1 (2.5)	10 (25)	**0.002**	0.56, **0.01**, **0.003**
4	9 (22.5)	9 (22.5)	12 (30)	0.67 **0.02**	− **0.008**, 0.65, **0.03**
8	18 (45)	7 (17.5)	16 (40)		
16	12 (30)	13 (32.5)	10 (25)	0.75	−
24	1 (2.5)	6 (15)	2 (5)	0.08	−
**Total analgesic tramadol requirement**					
0	0 (0)	0 (0)	5 (12.5)	**0.005**	−**0.02**, **0.02**
1	2 (5)	1 (2.5)	14 (35)	**0.001**	0.56 **0.001**, **0.001**
4	11 (27.5)	9 (22.5)	20 (50)	**0.02**	0.61 **0.04**, **0.01**
8	20 (50)	16 (40)	24 (60)	0.2	−
16	21 (52.5)	21 (52.5)	27 (67.5)	0.29	−
24	19 (47.5)	23 (57.5)	27 (67.5)	0.20	−
**Nausea and/or vomiting**					
0	0 (0)	1 (2.5)	7 (17.5)	**0.003**	0.31, **0.006**, **0.03**
1	5 (12.5)	5 (12.5)	9 (22.5)	0.37	−
4	6 (15)	9 (22.5)	10 (25)	0.52	−
8	8 (20)	12 (30)	10 (25)	0.59	−
16	7 (17.5)	12 (30)	11 (27.5)	0.39	−
24	7 (17.5)	13 (32.5)	11 (27.5)	0.3	−
**Complication**					
0	0 (0)	1 (2.5)	6 (15)	**0.009**	0.31 **0.01**, **0.04**
1	5 (12.5)	3 (7.5)	3 (7.5)	0.67	−
4	2 (5)	4 (10)	1 (2.5)	0.35	−
8	3 (7.5)	3 (7.5)	1 (2.5)	0.55	−
16	0 (0)	0 (0)	1 (2.5)	0.36	−
24	0 (0)	1 (2.5)	0 (0)	0.377	−
**Total analgesic tramadol requirement**	21 (52.5)	23 (57.5)	27 (67.5)	0.38	−
**Total antiemetic requirement**	10 (25)	12 (30)	13 (32.5)	0.75	−
**Nausea and/or vomiting**	10 (25)	12 (30)	12 (30)	0.85	−

* Chi-square test, ** z test; *p* < 0.05 is significant.

**Table 3:** Evaluation of instantaneous analgesic amounts between groups (n = 120).

	Bupivacaine (n = 40)	Bupivacaine +Dexmedetomidin (n = 40)	Bupivacaine + Dexametazon (n = 40)		
Hour	Mean ± SD	Mean ± SD	Mean ± SD	*p* Value *	*p* Value ** 1 vs. 2, 1 vs. 3, 2 vs. 3
**Instant analgesic tramadol amount (mg)**					
0	−	−	−	−	−
1	45 ± 0	40 ± −	37.2 ± 3.67	**0.04**	−**0.04**−
4	38.61 ± 3.33	38.06 ± 3.14	35.83 ± 3.68	0.16	−
8	39.56 ± 3.62	39.36 ± 5.37	36.37 ± 3.71	0.06 0.22	− −
16	40.67 ± 4.22	38.5 ± 6	37 ± 3.77		
24	37.5 ± -	42.67 ± 7.52	32.5 ± 3.53	0.27	−
**Total analgesic tramadol amount (mg)**					
0	−	−	−	**-**	−
1	45 ± 0	40 ± −	39.71 ± 13.21	0.86	−
4	39.77 ± 3.94	42.5 ± 14.39	47.65 ± 19.54	0.39	−
8	55.47 ± 19.81	41.13 ± 11.16	65.33 ± 26.04	**0.003**	0.11, 0.27, **0.002**
16	77.97 ± 31.31	55.57 ± 19.38	71.78 ± 34.96	**0.04**	**0.04**, 0.76, 0.14
24	80 ± 36.14	61.5 ± 27.38	74.19 ± 35.93	0.18	−

* One-Way ANOVA test, ** Post Hoc test-Tukey; *p* < 0.05 is significant. SD: Standard deviation.

**Table 4:** Assessment of visual analog score and satisfaction scale between groups (n = 120).

	Bupivacaine (n = 40)	Bupivacaine +Dexmedetomidin (n = 40)	Bupivacaine + Dexametazon (n = 40)		
Hour	Mean ± SD	Mean ± SD	Mean ± SD	*p* Value *	*p* Value ** 1 vs. 2, 1 vs. 3, 2 vs. 3
**VAS sitting position**					
0	0 (0–0) 0.05 ± 0.32	0 (0–0) 0.15 ± 0.48	0 (0–2) 0.85 ± 1.61	**0.005**	0.89, **0.001**, **0.005**
1	0.5 (0–2) 1.03 ± 1.21	0 (0–1) 0.7 ± 1.04	0 (0–2) 1.38 ± 1.76	0.33	−
4	2 (1–3) 2.28 ± 1.37	2 (1–2) 2.32 ± 1.47	2 (1–3) 2.42 ± 1.75	0.95 0.19	− −
8	2.5 (2–3) 3.02 ± 1.38	2 (2–3) 2.57 ± 1.01	2 (1–3) 2.85 ± 1.65		
16	2 (2–3) 2.88 ± 1.24	2 (2–3) 2.83 ± 1.1	2 (0.25–3) 2.25 ± 1.48	0.14	−
24	2 (2–3) 2.3 ± 0.68	2 (2–3) 2.57 ± 1.01	1 (1–3) 1.7 ± 1.2	**0.005**	0.45, **0.03**, **0.001**
**VAS supine position**					
0	0 (0–0) 0.05 ± 0.32	0 (0–0) 0.15 ± 0.48	0 (0–0) 0.85 ± 1.36	**0.001**	0.86, **0.001**, **0.001**
1	1 (0–2) 0.85 ± 0.97	0 (0–1) 0.63 ± 0.89	0 (0–3.75) 1.13 ± 1.32	0.29	−
4	2 (1–3) 2.07 ± 1.22	2 (1–3) 1.78 ± 1.12	2 (1–4) 1.98 ± 1.32	0.47	−
8	3 (2–4) 2.38 ± 1.03	3 (2–3) 2.18 ± 0.87	3 (2–4) 2.1 ± 1.23	0.43	−
16	3 (2–4) 2.25 ± 0.83	3 (2–4) 2.25 ± 0.92	2 (1–3.75) 1.73 ± 1.03	0.1	−
24	2 (2–3) 2.2 ± 0.72	3 (2–3) 2.28 ± 0.78	2 (1–3.5) 1.43 ± 1.05	**0.001**	0.92, **0.001**, **0.001**
**Satisfaction Scale**	3 (2–3) 2.73 ± 0.98	3 (3–4) 3.05 ± 0.78	3 (3–4) 3.3 ± 0.79	**0.02**	0.21, **0.009**, 0.39

* Kruskal–Wallis H test, ** Post Hoc test-Tukey; *p* < 0.05 is significant. Q1–Q3: 25%-75%. SD: Standard deviation, VAS: visual analog score.

## Data Availability

The datasets generated during and/or analyzed during the current study are available from the corresponding author upon reasonable request.

## References

[1] Sachdeva J., Sinha A. (2016). Randomized Controlled Trial to Study the Effect of Dexamethasone as Additive to Ropivacaine on Duration of Ultrasound-guided Transversus Abdominis Plane Block in Cesarean Section. Indian. J. Pain..

[2] Betran A.P., Ye J., Moller A.B., Zhang J., Gulmezoglu A.M., Torloni M.R. (2016). The Increasing Trend in Caesarean Section Rates: Global, Regional and National Estimates: 1990–2014. PLoS ONE.

[3] Betran A.P., Ye J., Moller A.B., Souza J.P., Zhang J. (2021). Trends and projections of caesarean section rates: Global and regional estimates. BMJ Glob. Health.

[4] Singla N., Garg K., Jain R., Malhotra A., Singh M.R., Grewal A. (2021). Analgesic efficacy of dexamethasone versus dexmedetomidine as an adjuvant to ropivacaine in ultrasound-guided transversus abdominis plane block for post-operative pain relief in caesarean section: A prospective randomised controlled study. Indian. J. Anaesth..

[5] Neethirajan S.G.R., Kurada S., Parameswari A. (2020). Efficacy of dexmedetomidine as an adjuvant to bupivacaine in ultrasound-guided transverse abdominis plane block for laparoscopic appendicectomy: A randomised controlled study. Turk. J. Anaesthesiol. Reanim..

[6] Storti E., Vaccari S., Bruno R., Ricchi A., Buffagni J., Neri I. (2018). Guidelines for management of analgesics after caesarean section: Cognitive survey. Int. J. Womens Health Wellness.

[7] Rafi A.N. (2001). Abdominal field block: A new approach via the lumbar triangle. Anaesthesia.

[8] Vincent M., Mathieu O., Nolain P., Menace C., Khier S. (2020). Population Pharmacokinetics of Levobupivacaine During Transversus Abdominis Plane Block in Children. Ther. Drug Monit..

[9] Thakur S., Sharma A., Kaushal S., Sharma A., Sharma N., Thakur P.S. (2023). Comparison of Clonidine with Bupivaicaine vs Plain Bupivaicaine in Transversus Abdominis Plane (TAP) Block in Women Undergoing Cesarean Delivery Under Spinal Anesthesia: Randomized Clinical Trial. J. Pharm. Bioallied Sci..

[10] Sujata N., Hanjoora V. (2014). Pain control after cesarean birth–What are the options. J. Gen. Pract..

[11] Jadon A., Jain P., Chakraborty S., Motaka M., Parida S.S., Sinha N., Agrawal A., Pati A.K. (2018). Role of ultrasound guided transversus abdominis plane block as a component of multimodal analgesic regimen for lower segment caesarean section: A randomized double blind clinical study. BMC Anesthesiol..

[12] Abdallah F.W., Laffey J.G., Halpern S.H., Brull R. (2013). Duration of analgesic effectiveness after the posterior and lateral transversus abdominis plane block techniques for transverse lower abdominal incisions: A meta-analysis. Br. J. Anaesth..

[13] Collins J.B., Song J., Mahabir R.C. (2013). Onset and duration of intradermal mixtures of bupivacaine and lidocaine with epinephrine. Can. J. Plast. Surg..

[14] Sivakumar R.K., Panneerselvam S., Cherian A., Rudingwa P., Menon J. (2018). Perineural vs. intravenous dexmedetomidine as an adjunct to bupivacaine in ultrasound guided fascia iliaca compartment block for femur surgeries: A randomised control trial. Indian. J. Anaesth..

[15] Ramya Parameswari A., Udayakumar P. (2018). Comparison of efficacy of bupivacaine with dexmedetomidine versus bupivacaine alone for transversus abdominis plane block for post-operative analgesia in patients undergoing elective caesarean section. J. Obstet. Gynecol. India.

[16] Zhang P., Liu S., Zhu J., Rao Z., Liu C. (2019). Dexamethasone and dexmedetomidine as adjuvants to local anesthetic mixture in intercostal nerve block for thoracoscopic pneumonectomy: A prospective randomized study. Reg. Anesth. Pain Med..

[17] Maniaci A., Lechien J.R., Calvo-Henriquez C., Iannella G., Leigh S., Ingrassia A., Merlino F., Bannò V., Cocuzza S., La Mantia I. (2022). Long-term stability of outcomes of endoscopic surgery for rhinogenic contact point headache (Sluder’’s neuralgia). Am. J. Otolaryngol..

[18] Teodoro M., Scibilia G., Lomeo E., Pecorino B., Galia A., Scollo P. (2019). Carbon dioxide laser as a new valid treatment of lichen sclerosus. Clin. Exp. Obstet. Gynecol..

[19] Association W.M. (2013). World Medical Association Declaration of Helsinki: Ethical principles for medical research involving human subjects. JAMA.

[20] Huskisson E.C. (1974). Measurement of pain. Lancet.

[21] Norman G. (2010). Likert scales, levels of measurement and the “laws” of statistics. Adv. Health Sci. Educ. Theory Pract..

[22] McDonnell J.G., Curley G., Carney J., Benton A., Costello J., Maharaj C.H., Laffey J.G. (2008). The analgesic efficacy of transversus abdominis plane block after cesarean delivery: A randomized controlled trial. Anesth. Analg..

[23] Benyamin R., Trescot A.M., Datta S., Buenaventura R.M., Adlaka R., Sehgal N., Glaser S.E., Vallejo R. (2008). Opioid complications and side effects. Pain Physician.

[24] Imani F., Rahimzadeh P., Faiz H.-R., Abdullahzadeh-Baghaei A. (2018). An evaluation of the adding magnesium sulfate to ropivacaine on ultrasound-guided transverse abdominis plane block after abdominal hysterectomy. Anesthesiol. Pain Med..

[25] Scibilia G., Pecorino B., Pagano I., Scollo P. (2017). Case report: Laparoscopic uterovaginal anastomosis for congenital isolated cervical agenesis. J. Minim. Invasive Gynecol..

[26] Cummings K.C., Napierkowski D., Parra-Sanchez I., Kurz A., Dalton J., Brems J., Sessler D. (2011). Effect of dexamethasone on the duration of interscalene nerve blocks with ropivacaine or bupivacaine. Br. J. Anaesth..

[27] Gupta A., Gupta A., Yadav N. (2019). Effect of dexamethasone as an adjuvant to ropivacaine on duration and quality of analgesia in ultrasound-guided transversus abdominis plane block in patients undergoing lower segment cesarean section-A prospective, randomised, single-blinded study. Indian J. Anaesth..

[28] Akkaya A., Yildiz I., Tekelioglu U., Demirhan A., Bayir H., Ozlu T., Bilgi M., Kocoglu H. (2014). Dexamethasone added to levobupivacaine in ultrasound-guided tranversus abdominis plain block increased the duration of postoperative analgesia after caesarean section: A randomized, double blind, controlled trial. Eur. Rev. Med. Pharmacol. Sci..

[29] Talebi G., Moayeri H., Rahmani K., Nasseri K. (2021). Comparison of three different doses of dexmedetomidine added to bupivacaine in ultrasound-guided transversus abdominis plane block; a randomized clinical trial. Anesthesiol. Pain Med..

[30] Ammar A.S., Mahmoud K.M. (2012). Effect of adding dexamethasone to bupivacaine on transversus abdominis plane block for abdominal hysterectomy: A prospective randomized controlled trial. Saudi J. Anaesth..

[31] Thakur J., Gupta B., Gupta A., Verma R.K., Verma A., Shah P. (2019). A prospective randomized study to compare dexmedetomidine and dexamethasone as an adjunct to bupivacaine in transversus abdominis plane block for post-operative analgesia in caesarean delivery. Int. J. Reprod. Contracept. Obstet. Gynecol..

[32] Sinha J., Pokhriyal A.S., Asthana V., Nautiyal R. (2023). Dexmedetomidine vs Dexamethasone as an Adjuvant to Levobupivacaine in Ultrasound-Guided Transversus Abdominis Plane Block for Postoperative Analgesia in Patients Undergoing Total Abdominal Hysterectomies. Anesthesiol. Pain Med..

